# Mental Disorders, Social Media Addiction, and Academic Performance in Romanian Undergraduate Nursing Students

**DOI:** 10.3390/jcm13154475

**Published:** 2024-07-31

**Authors:** Liliana David, Abdulrahman Ismaiel, Paul Foucambert, Daniel Corneliu Leucuta, Stefan-Lucian Popa, Mihaela Fadgyas Stanculete, Dan L. Dumitrascu

**Affiliations:** 1 2nd Department of Internal Medicine, “Iuliu Hatieganu” University of Medicine and Pharmacy, 400006 Cluj-Napoca, Romania; lilidavid2007@yahoo.com (L.D.); abdulrahman.ismaiel@yahoo.com (A.I.); paul.foucambert@gmail.com (P.F.); popa.stefan@umfcluj.ro (S.-L.P.); ddumitrascu@umfcluj.ro (D.L.D.); 2 Department of Medical Informatics and Biostatistics, “Iuliu Hatieganu” University of Medicine and Pharmacy, 400349 Cluj-Napoca, Romania; 3 Department of Neurosciences, “Iuliu Hatieganu” University of Medicine and Pharmacy, 400006 Cluj-Napoca, Romania; mihaelastanculete@yahoo.com; 4 Institute of Advanced Studies in Science and Technology, Babes-Bolyai University, 400347 Cluj-Napoca, Romania

**Keywords:** anxiety, depression, mental disorders, social media addiction, internet addiction

## Abstract

**Introduction**: We aimed to evaluate social media addiction in Romanian undergraduate nursing students and its association with academic performance, depression, and anxiety. **Methods:** We used a cross-sectional online survey to collect data among nursing undergraduate students enrolled at the University of Medicine and Pharmacy “Iuliu Hatieganu”, Cluj-Napoca, Romania. The Social Media Addiction Scale-Student Form (SMAS-SF), Beck Depression Inventory (BDI), and State-Trait Anxiety Inventory (STAI) were used to assess social media addiction, depression, and anxiety, respectively. **Results**: A total of 90 nursing students participated in the study, of which 82 (91.1%) were females and 81 (90%) were of Romanian ethnicity. The median age of participants was 21 years (18–40). Males showed higher BDI total scores, SMAS-SF total scores, and STAI scale A state anxiety compared to females, and females showed higher STAI scale A trait anxiety compared to males, although these results were not statistically significant. Also, we found no significant difference in these variables between participants from different study years. Participants with no or mild depression had a significantly higher academic performance (*p*-value = 0.001), lower SMAS-SF (*p*-value = 0.004), and lower STAI scores (*p*-value < 0.001) compared to participants with borderline, moderate, or severe depression after performing multivariate regression analysis. **Conclusions**: Our study demonstrated that depression was significantly associated with social media addiction, anxiety, and lower academic performance in Romanian undergraduate nursing students.

## 1. Introduction

Social media has integrated itself into people’s daily activities [1]. In 2019, one-third of all people worldwide and two-thirds of all internet users were using social media platforms. The vast implementation of these platforms—which include Facebook, Instagram, Twitter, Snapchat, YouTube, and WhatsApp—has dramatically changed the ways of communicating, accessing information, and meeting people [2]. We spend, on average, two hours per day interacting and updating content on social media, with around 500,000 Snapchat photos and tweets being shared every minute [3]. Younger people generally tend to spend more time on these platforms than older individuals, with Twitter and Instagram being now more valued by adolescents [2,4]. In the USA, 93% of the population aged 15 to 17 have mobile internet access, and 45% of US teenagers report that they are nearly constantly online [4,5].

Social media has become an important part of young people’s lives [1,5]. On the other hand, the incidence of depression, anxiety, and suicidality in adolescent populations has substantially increased over the last 10–15 years [5]. This correlation has raised concerns about the impact of the internet and social media on young adults’ mental health, and their association has been increasingly studied in recent years [4,6]. Social media use has been shown to have a negative impact on psychological health, interpersonal relationships, and private life [7]. In addition, it can lead to social media addiction, which has been associated with depression [7,8,9], stress [10], anxiety [9,11], social anxiety [12], low self-esteem [13], and burnout [14]. Social media addiction has also demonstrated negative effects on sleep quality in students, which can, in turn, lead to a reduction in their school performance and energy levels [15]. Possible reasons/mechanisms behind increasing social media addiction include social pressure, social comparison, and social reward [16]. Lack of inhibitory control, a feature that develops through adolescence until adulthood, is also associated with addiction behaviors (e.g., drugs, gambling), including social media addiction [17,18,19].

Social media is becoming progressively more popular among nursing students [20,21]. Their use has helped students improve their understanding of communication, ethics, and professionalism and can assist them in building professional networks, enhancing their knowledge, and increasing involvement in nursing communities [20,21]. While social media use in nursing students demonstrated several advantages, addiction to social media in this population presents considerable risks and might negatively impact their mental health and university achievements. Previous studies conducted on the Romanian population explored the relationship between social media addiction and depression [22,23], anxiety [23], social anxiety [24], and happiness [25], but no previous study investigated its association with academic performance in nursing students. Due to this lack of knowledge, we aimed to assess social media addiction among Romanian undergraduate nursing students and its association with academic performance and common mental disorders, including depression and anxiety. We hypothesized that social media addiction in undergraduate nursing students could be associated with depression, anxiety, and lower academic performance.

## 2. Methods

### 2.1. Study Design

We conducted a cross-sectional research design to assess social media addiction in Romanian nursing students and its relationship with academic performance, depression, and anxiety.

We used an online survey among nursing undergraduate students from the University of Medicine and Pharmacy “Iuliu Hatieganu” of Cluj-Napoca, Romania. The study was completed in October 2022 and aimed at reaching nursing students of all years. Students enrolled at the University in the first year of studies and onwards and responding to the survey were included in the study. The sample size corresponds to the number of students who completed the survey. The questionnaires were sent using an online link, and data were automatically collected using Google Forms. No data were missing as all obligatory fields had to be completed to submit the questionnaires.

### 2.2. Study Measures

The self-administered questionnaire comprised basic demographic questions, including age, sex, year of study, and ethnicity. The average academic performance in the previous year was collected using self-reported statements from the students who completed their first university year. Grades range from 1 to 10; a score lower than five represents failing the exam, while higher values mean better grades. Social media addiction, depression, and anxiety were evaluated using validated measurement tools.

We evaluated social media addiction using the Romanian form of the Social Media Addiction Scale-Student Form (SMAS-SF). This questionnaire consists of 29 questions and four subdimensions, with possibly obtained scores ranging from 29 to 145 [26]. No information regarding time spent, equipment used (e.g., PC, smartphone), or type of social media (e.g., Facebook, WhatsApp) was gathered.

We assessed the presence of depression using the Beck Depression Inventory (BDI), a self-assessment tool consisting of 21 items evaluating symptoms of depression according to the diagnostic criteria of depressive disorders included in the Diagnostic and Statistical Manual of Mental Disorders—4th Edition of the American Psychiatric Association (DSM-IV) [27,28]. The score can range from 0 to 63 and is obtained by adding the scores of each item [29].

We evaluated anxiety in study participants with the State-Trait Anxiety Inventory (STAI), a diagnostic tool that contains scales that measure temporary anxiety, known as state anxiety (A-State), and long-term anxiety, known as trait anxiety (A-Trait) [30,31].

Results were reported as median (with interquartile range [IQR]), difference (with 95% confidence interval [CI]), and *p*-values.

### 2.3. Statistical Analysis

The R software environment for statistical computing and graphics version 4.0.2 (R Foundation for Statistical Computing, Vienna, Austria) was used to perform the statistical analysis. Among the R packages used, there were psych and car, besides the standard installed ones. We used frequencies and percentages to report categorical data. Concerning continuous data, we reported normally distributed data as mean (standard deviation (SD)) and non-normally distributed data as median (interquartile range (IQR)). Quantile–quantile plots were used to determine the normality of the distribution of the data. The *t*-test for independent samples of normally distributed data was used to compare the clinical characteristics of the study population according to the categorized groups. In addition, we used the Wilcoxon rank-sum test for non-normally distributed data, and the Chi-square and Fisher exact tests were employed for categorical data. In case the expected frequencies were low, we used the Fisher exact test; otherwise, the Chi-square tests were used. To assess the association between social media addiction, depression, and academic performance while controlling for age, we carried out a multivariate linear regression analysis. The assumptions of residuals normality, multicollinearity, and linearity of predictors with the dependent variables were checked, with residuals quantile-quantile plots, variable inflation factors, and component+residual plots. The regression results were reported as model coefficients, 95% confidence interval (CI), and *p*-value.The internal consistency of each measure (SMAS-SF, BDI), as well as the subdomains of STAI (state and trait), were assessed with raw and standardized Cronbach’s alpha, with 95% confidence intervals, and Guttman’s Lambda 6. Furthermore, we assessed the average inter-item correlations, as well as the reliability, by checking the effect on Cronbach’s alpha if any of the items were dropped. For all analyses, two-sided statistical tests were performed. We considered a *p*-value of <0.05 to be statistically significant.

## 3. Results

Among the 90 undergraduate nursing students participating in the research, 82 students (91.1%) were females, 81 (90%) students were of Romanian ethnicity, and their median age was 21 years [19,20,21,22]. The study participants consisted of 32 (35.6%) first-year students, 22 (24.4%) second-year students, 2 (2.2%) third-year students, and 34 (37.8%) fourth-year students (Table 1).

The SMAS-SF total score was found to be 73 (65–80.75), the BDI total score was 8 (3–14), the STAI scale A state anxiety score was 39.5 (32–48.75), the STAI scale A trait anxiety score was 47 (42–54.75), and the academic performance in the previous year was 8 (8–9), as shown in Table 2.

According to the BDI classification, 16 (17.78%) students had mild depression, 6 (6.67%) students had borderline depression, 6 (6.67%) had moderate depression, 2 (2.22%) had severe depression, and 4 (4.44%) had extreme depression, as shown in Table 3.

We assessed the internal consistency of the measurement instruments. Concerning the SMAS-SF questionnaire, the raw and standardized Cronbach’s alpha value of 0.88 (95% CI 0.84–0.91) indicates excellent internal consistency. The additional statistics support the reliability of the scale, with acceptable average inter-item correlations and a high Guttman’s Lambda 6 (0.94). The narrow confidence interval further confirms the precision of the reliability estimate. The item statistics reveal that each item has a moderate to strong correlation with the total score, suggesting that all items contribute meaningfully to the measurement of the underlying construct.

Concerning the BDI questionnaire, the raw and standardized Cronbach’s alpha value of 0.95 (95% CI 0.93–0.96) indicates excellent internal consistency. The additional statistics support the reliability of the scale, with high average inter-item correlations and Guttman’s Lambda 6 values (0.97). The narrow confidence interval further confirms the precision of the reliability estimate. The item statistics reveal that each item has a moderate to strong correlation with the total score, suggesting that all items contribute meaningfully to the measurement of depression.

The STAI scale has two parts. The raw and standardized Cronbach’s alpha values were 0.95 (95% CI 0.94–0.97) for the state STAI subscale and 0.93 (95% CI 0.91–0.95) for the trait subscale, indicating excellent internal consistency. The additional statistics support the reliability of the scale, with high average inter-item correlations and Guttman’s Lambda 6 values (0.97 for state STAI subscale and 0.95 for trait STAI subscale). The narrow confidence interval further confirms the precision of the reliability estimate.

We evaluated the average academic performance within the previous year in relation to age, SMAS-SF total score, and BDI total score, and no significant association was found (*p*-value ≥ 0.05) (Table 4).

Female participants were found to have higher academic grades in the previous year and higher STAI scale A trait anxiety compared to males. Male participants had greater BDI total scores, SMAS-SF total scores, and STAI scale A state anxiety compared to females. However, none of these results were statistically significant (*p*-value ≥ 0.05) (Table 5).

The academic performance in the previous year was significantly different among students based on the year of study (*p*-value = 0.047). The BDI total score, SMAS-SF total score, STAI scale A—state anxiety score, and STAI scale A—trait anxiety score did not demonstrate a significant difference between different study years (*p*-value ≥ 0.05) (Table 6).

Compared to students with borderline, moderate, and severe depression, participants having no or mild depression demonstrated significantly higher average academic performance in the previous year (*p*-value = 0.001), lower SMAS-SF total score (*p*-value = 0.004), lower STAI scale A—state anxiety score (*p*-value < 0.001), and lower STAI scale A—trait anxiety score (*p*-value < 0.001). The study year and age of students did not show a significant difference based on depression severity (*p*-value ≥ 0.05) (Table 7).

## 4. Discussion

Due to the steady increase in social media use among nursing students, its potential consequences on their mental health and school performance have gained more attention in recent years [32,33]. Although several observational studies have discussed social media use in nursing students in association with academic achievements [32,34,35] and social media use in nursing students in association with their mental health [36,37], no study combining both of these aspects was previously realized. Moreover, none of these studies took place in Romania. To the best of our knowledge, this is the first cross-sectional study to evaluate social media addiction in Romanian undergraduate nursing students and its relationship with academic performance, depression, and anxiety. The present study demonstrated that depression was significantly associated with social media addiction, anxiety, and lower academic performance in Romanian undergraduate nursing students.

Several points need to be further discussed. Firstly, we used the BDI to detect the presence of depression in study participants and to stratify its severity, hence providing a trustworthy way of assessing depression. Indeed, previous studies have consistently reported its accuracy, validity, and reliability in detecting depression in children, adolescents, and adults [38,39,40]. The BDI has been proven to be an effective screening tool in primary care settings, being helpful not only to healthy patients but also to patients with chronic diseases and cancers [38,41,42]. We were, therefore, able to detect depression accurately in nursing students, regardless of their age and overall health. Secondly, the STAI was used to evaluate anxiety in participants. The STAI scales have demonstrated consistency and reliability in measuring trait and state anxiety in patients from various medical and psychiatric settings [43,44,45,46]. Moreover, these scales have been used to evaluate state–trait anxiety in student populations and have shown satisfactory reliability and validity [47,48]. Hence, we selected a valid and accurate measurement tool that would be adequate for the population we studied. Thirdly, we evaluated social media addiction among study participants with the SMAS-SF. Its Romanian version was shown to be a valid and reliable measurement tool that accurately detects social media addiction in undergraduate students, therefore being especially suitable to our study [26].

Our study revealed that undergraduate nursing students having borderline, moderate, or severe depression according to the BDI classification demonstrated higher addiction to social media, higher rates of trait and state anxiety, and lower academic performance compared to students with no or mild depression. Our findings are consistent with previous studies, which have recently demonstrated the harmful effects of excessive social media use on mental health and its correlation with depression among university students [49,50]. Indeed, problematic social media use seems to reduce perceived social support, increase social media violence, and increase loneliness, negatively affecting happiness and mental health and thereby increasing depressive symptoms [51,52]. Prolonged social media use can negatively affect relationships with friends and family by reducing face-to-face interactions, which are important to well-being. In addition, the excessive use of social media, especially at nighttime, can lead to pre-sleep hyperarousal and decreased sleep duration. In turn, sleep deprivation can negatively affect mood and cognitive function, therefore promoting the development of depression [53].

Moreover, it is not surprising to observe an association between depression and anxiety in study participants. Indeed, these are common conditions that can affect up to 25% of general practice patients, and their relationship has been well-established by previous research over the years [54,55]. It was shown that 85% of patients with depression have comorbid anxiety, and 90% of patients with anxiety disorders have concurrent depression [55]. The comorbidity of those disorders is associated with treatment resistance, increased risk of recurrence, higher usage of medical resources, greater risk of suicide, and overall worse outcomes [56]. This highlights the importance of a careful and comprehensive approach when managing patients suffering from concurrent anxiety and mood disorders [57]. Our study supports previous research that had shown a high prevalence of depression and anxiety in nursing students [58], further emphasizing the need for a prompt and mindful psychiatric assessment in these populations whenever there is suspicion of a mood or anxiety disorder.

University students usually experience a critical transitory time between adolescence and adulthood, a particularly stressful period in one’s life together with other factors such as distance from home, social pressure, professional projects, and exam stress, put university students at particular risk of depression and anxiety, both being frequently seen in those populations [59,60]. Both depression and anxiety have been shown to affect cognitive function, which in turn can negatively impact academic performance [60]. Previous studies have demonstrated a correlation between depression and poorer academic results in university students [60,61,62,63], findings that are further supported by our current study. Students suffering from depression may show a loss of interest in their studies, reduced concentration, loss of energy, and sleep disturbances, all of which can negatively affect their performance at school [62]. It was shown, however, that students being treated for depression had a significant improvement in their academic performance and were obtaining grades comparable to the ones who were not suffering from it [61]. This, therefore, emphasizes the importance of proper and timely diagnosis of depression in university students, as well as its prompt treatment.

Finally, we found no significant difference in depression and anxiety between male and female participants, as opposed to previous studies that demonstrated a higher prevalence of depression and anxiety in young adult women compared to men [64,65,66,67,68]. We also did not find a significant difference in social media addiction between both sexes, unlike previous research, which demonstrated that females had a higher rate of social media addiction (while males had higher rates of video game addiction) [69,70]. We found no significant difference in academic performance between male and female nursing students, a finding that showed inconsistent results in previous research: some data showed higher academic performance in female nursing students compared to males [71], while other data did not show any significant difference between both sexes [72]. We may have failed to detect such differences due to the limited number of subjects used in our analysis.

Our study has several limitations. The cross-sectional design of our study, although permitting us to demonstrate correlation, cannot serve as a way of establishing causality between the presented associations. In addition, due to the moderate sample size of our study, we could not perform subgroup analysis, therefore limiting our evaluation of the assessed variables in sex, study year, and ethnicity subpopulations. Moreover, our study was carried out in a single center on a very specific sample (Romanian undergraduate nursing students), potentially preventing the generalizability of our results to other populations. The lack of data on foreign and off-site students limits our ability to fully understand how these factors influence social media addiction within this specific population. Including these groups in future research could help disentangle various socio-cultural and environmental aspects that may affect the propensity for social media addiction. Finally, due to our study using an online survey and having a cross-sectional design, there is a risk of selection bias, mainly in the form of sampling bias and non-response bias [73].

## 5. Conclusions

Our study demonstrated that depression was significantly associated with social media addiction, anxiety, and lower academic performance among Romanian undergraduate nursing students. Our results, while confirming previous research, bring a new and unique way of testing those variables in combination. We aimed to highlight these associations, understand their mechanism, and emphasize their implications for the mental health, academic life, and psychiatric management of nursing students.

## Figures and Tables

**Table 1:** Characteristics of study participants.

Characteristics		Participants (*n* = 90), Number (%)
**Sex**	Males	8 (8.9%)
	Females	82 (91.1%)
**Study year**	1st year	32 (35.6%)
	2nd year	22 (24.4)
	3rd year	2 (2.2%)
	4th year	34 (37.8%)
**Ethnicity**	Romanian	81 (90%)
	Hungarian	7 (7.8%)
	Roma	1 (1.1%)
	Other	1 (1.1%)

**Table 2:** Participants’ BDI total score, SMAS-SF total score, STAI scores, age, and academic performance in the previous year.

Characteristic	Participants (*n* = 90)
BDI Beck depression index total score, median (IQR)	8 (3–14)
SMAS-SF total score, median (IQR)	73 (65–80.75)
STAI scale A—state anxiety, median (IQR)	39.5 (32–48.75)
STAI scale A—trait anxiety, median (IQR)	47 (42–54.75)
Age, median (IQR)	21 (19–22)
Academic performance in the previous year, median (IQR)	8 (8–9)

**Table 3:** Distribution of depression severity according to the BDI classification.

BDI Classification	Nr.	%
Normal	56.00	62.22
Mild	16.00	17.78
Borderline	6.00	6.67
Moderate	6.00	6.67
Severe	2.00	2.22
Extreme	4.00	4.44

**Table 4:** Multivariate linear regression model of the average academic performance in the previous year in relation to age, SMAS-SF total score, and BDI total score.

Characteristics	B	(95% CI)	*p*-Value
Age	0.01	(−0.05–0.06)	0.834
SMAS-SF total score	0	(−0.02–0.01)	0.574
BDI Beck depression index total score	−0.01	(−0.03–0)	0.147

**Table 5:** BDI total score, academic performance in the previous year, SMAS-SF total score, and STAI scores based on the sex of participants.

Characteristics	Females (*n* = 82)	Males (*n* = 8)	*p*-Value
BDI Beck depression index total score, median (IQR)	8 (3–14)	10 (1.5–17.5)	0.865
Academic performance in the previous year, median (IQR)	8 (8–9)	8 (7.5–8)	0.275
SMAS-SF total score, median (IQR)	72.5 (65–80)	78.5 (71.75–89)	0.192
STAI scale A—state anxiety, median (IQR)	39.5 (32–48)	39.5 (35.5–53.5)	0.702
STAI scale A—trait anxiety, median (IQR)	47 (42–54.75)	45.5 (35.75–53.5)	0.469

**Table 6:** BDI total score, academic performance in the previous year, SMAS-SF total score, and STAI scores based on the study year of participants.

Characteristics	Year 1 (*n*= 32)	Year 2 (*n* = 22)	Year 3 (*n* = 2)	Year 4 (*n* = 34)	*p*-Value
BDI Beck depression index total score, median (IQR)	8 (4.75–15)	7.5 (2–13.5)	2 (1–3)	8.5 (2–13.75)	0.398
Academic performance in the previous year, median (IQR)	8.5 (8.25–8.75)	8 (8–8)	9 (9–9)	8 (8–9)	0.047
SMAS-SF total score, median (IQR)	74.5 (66.75–81.5)	74 (65–81)	71.5 (69.25–73.75)	71 (64.25–79)	0.732
STAI scale A—state anxiety, median (IQR)	37.5 (30.5–49.25)	42 (31.25–47)	32.5 (31.75–33.25)	39.5 (34–49.75)	0.618
STAI scale A—trait anxiety, median (IQR)	46.5 (42.75–60)	47.5 (40–54)	45 (40.5–49.5)	47.5 (42–53)	0.94

**Table 7:** Study year, academic performance in the previous year, SMAS-SF total score, STAI scores, and age based on the severity of depression.

Characteristics	Borderline/Moderate/Severe (*n* = 18)	Normal/mild (*n* = 72)	*p*-Value
Study year, median (IQR)	2 (1–4)	2 (1–4)	0.634
Academic performance in the previous year, median (IQR)	8 (7–8)	8 (8–9)	0.001
SMAS-SF total score, median (IQR)	81 (74.25–86)	71 (64–77.25)	0.004
STAI scale A—state anxiety, median (IQR)	54 (44.25–59)	37 (31–44)	<0.001
STAI scale A—trait anxiety, median (IQR)	60 (53.5–67.25)	45 (40–52)	<0.001
Age, median (IQR)	21 (19–22.75)	21 (19–22)	0.617

## Data Availability

Further information can be obtained by contacting the corresponding author.
